# Implementing Clinical Pharmacogenomics in the Classroom: Student Pharmacist Impressions of an Educational Intervention Including Personal Genotyping

**DOI:** 10.3390/pharmacy6040115

**Published:** 2018-10-23

**Authors:** Amber Frick, Cristina Benton, Oscar Suzuki, Olivia Dong, Rachel Howard, Hijrah El-Sabae, Tim Wiltshire

**Affiliations:** Eshelman School of Pharmacy, University of North Carolina, Chapel Hill, NC 27599, USA; santosc@email.unc.edu (C.B.); osuzuki@email.unc.edu (O.S.); odong@email.unc.edu (O.D.); howardrm@email.unc.edu (R.H.); hijrah_elsabae@unc.edu (H.E.-S.); timw@unc.edu (T.W.)

**Keywords:** personalized medicine, pharmacogenomics, genotyping

## Abstract

Pharmacogenomics provides a personalized approach to pharmacotherapy by using genetic information to guide drug dosing and selection. However, partly due to lack of education, pharmacogenomic testing has not been fully implemented in clinical practice. With pharmacotherapy training and patient accessibility, pharmacists are ideally suited to apply pharmacogenomics to patient care. Student pharmacists (*n* = 222) participated in an educational intervention that included voluntary personal genotyping using 23andMe. Of these, 31% of students completed both pre- and post-educational interventions to evaluate their attitudes and confidence towards the use of pharmacogenomics data in clinical decision making, and 55% of this paired subset obtained personal genotyping. McNemar’s test and the Wilcoxon signed-rank test were used to analyze responses. Following the educational intervention, students regardless of genotyping were more likely to recommend personal genotyping (36% post-educational intervention versus 19% pre-educational intervention, *p* = 0.0032), more confident in using pharmacogenomics in the management of drug therapy (51% post-educational intervention versus 29% pre-educational intervention, *p* = 0.0045), and more likely to believe that personalized genomics would have an important role in their future pharmacy career (90% post-educational intervention versus 51% pre-educational intervention, *p* = 0.0072) compared to before receiving the educational intervention. This educational intervention positively influenced students’ attitudes and confidence regarding pharmacogenomics in the clinical setting. Future studies will examine the use of next-generation sequencing assays that selectively examine pharmacogenes in the education of student pharmacists.

## 1. Introduction

Pharmacogenomics is the study of the relationship between genetic biomarkers and variation of individual drug response, metabolism, and transport. The application of clinical pharmacogenomics can provide a patient-personalized approach to drug therapy by using genetic information to guide drug dosing and selection. Using patient pharmacogenetic information, healthcare providers can choose drugs that are more likely to be efficacious, avoid side effects, optimize patient-specific doses, and/or determine the need for closer monitoring. The US Food and Drug Administration lists more than 140 therapeutic products with pharmacogenomic information in which specific action could be taken based on biomarker information [1]. Various evidence-based, peer-reviewed guidelines on clinical pharmacogenomics [2,3] also exist to assist health providers in patient drug management. As the most accessible healthcare providers and medication experts, pharmacists are well-suited to direct and deliver pharmacogenomics-based patient care [4,5,6,7].

Substantial scientific progress has been made in the understanding between genetic variation and variability of drug response and effect; however, pharmacogenomic testing has not been fully implemented in clinical practice. Potential barriers include cost-effectiveness and reimbursement, ethical concerns, and required educational and equipment infrastructure. Healthcare professionals, including pharmacists, report a lack of confidence in applying clinical pharmacogenomics despite the belief that it is important [4,8]. Opportunities to improve patient care based on pharmacogenomic-guided recommendations are missed due to insufficient clinical training and knowledge of how to translate genetic test results into clinical action based on currently available evidence. While 92% of US pharmacy schools have incorporated pharmacogenomics into their curricula [9], only 17% of practicing pharmacists reported their understanding of pharmacogenomics as “excellent”, “very good”, or “good” [10]. This knowledge gap is a significant barrier to widespread implementation of pharmacogenomic-based medicine, and exposing student pharmacists early in the curriculum with in-depth education and hands-on application of pharmacogenomics may further increase student comfort and foster a positive perspective towards pharmacogenomics [11,12]. Established core competencies outlined by the American Association of Colleges of Pharmacy (AACP) [13], Genetics/Genomics Competency Center (G2C2) [14] and the 2014 American Society of Health-System Pharmacists (ASHP) statement address the fundamental responsibility pharmacists have to ensure pharmacogenomics testing is performed as needed and that results are used for medication therapy optimization [15]. These competencies may help strengthen the focus of pharmacogenomic education in Doctor of Pharmacy (PharmD) program curricula and advance the role of the profession.

The purpose of this study was to evaluate the impact of an educational intervention including personal genotyping on student pharmacists’ attitudes and self-efficacy towards clinical pharmacogenomics. This study was a continuation of a previous study [16] and combines the results of both years. It was hypothesized that personalized genotyping would lead to more real-life understanding of the benefits of personal genomics and clinical acceptance.

## 2. Materials and Methods

Subjects included two classes of second-year pharmacy students attending the UNC Eshelman School of Pharmacy during Spring 2015 and Spring 2016 semesters. The student cohort included individuals from Chapel Hill and satellite Asheville campuses enrolled in a 15-week course designated as Pharmaceutical Care Lab (PCL), which was required as part of the PharmD curriculum. This lab-based course allowed students the opportunity to practice communication and develop competencies in skills (e.g., compounding, medication and drug-delivery device counseling and demonstration, and completion of patient cases). Students provided informed consent to participate in this study; participation was voluntary and did not influence their coursework grade. The UNC Institutional Review Board determined that this study was exempt from review.

The PCL consisted of a once weekly 1-h large group lecture with attendance from all second-year pharmacy students and weekly 4-h small group sessions consisting of 8–10 students per group. All students were led by clinical laboratory instructors (e.g., pharmacy residents and clinicians) in the large and small group sessions. These instructors were all practicing pharmacists but had various backgrounds in terms of practice setting (e.g., community, ambulatory care, and hospital) and previous exposure to and interest in pharmacogenomics. None of the instructors had specialized training in pharmacogenomics (e.g., pharmacogenomics residency or certificate program).

Educational intervention materials consisted of (1) a PowerPoint presentation with background information about pharmacogenomics; (2) an evidence-based educational video through 23andMe to help students understand basic methodologies employed in pharmacogenomics tests; (3) a demonstration to guide students through the logistics of sample acquisition; and (4) a pre-testing consultation using the 23andMe platform during a large class session on week 8 of the course. On week 11 in small group sessions, students received (5) small group case reviews with hands-on training for managing drug therapies based on the pharmacogenomic results from a demo 23andMe test and (6) exposure to real-world patient case scenarios through various counseling exercises. Further details describing the timing of the various components of the educational intervention are described in Appendix A.

An anonymous electronic survey was administered during week 8 of the PCL course before the introductory pharmacogenomic presentation. The same survey was conducted with additional questions upon completion of the pharmacogenomic lecture series in week 15 with discussion of how to interpret their personal 23andMe results in a large class session. The survey was adopted from prior published surveys on medical and graduate students’ attitudes towards genomics and personalized medicine [17,18,19] and modified to target student pharmacists. The survey gathered student demographics and assessed enrollment in previous genetics courses. Additionally, the survey assessed student pharmacists’ professional and personal attitudes and self-efficacy related to clinical pharmacogenomics and personalized genome testing. Survey questions prompted individuals to respond to their level of agreement with statements using a five-point Likert scale (i.e., strongly agree, agree, neither agree nor disagree, disagree, and strongly disagree), yes/no, or yes/no/maybe answers. Survey responses were linked using the same alphanumeric code for the pre- and post-intervention surveys to maintain student anonymity.

The pharmacy students were offered voluntary personal genomic testing by the direct-to-consumer 23andMe test (Mountain View, CA, USA) at a discounted price of $30.00. Funding from the UNC Center for Pharmacogenomics and Individualized Therapy (CPIT) was provided. Students were informed about the nature of their participation, including personal health information and potential risks beforehand. Willing participants could obtain the 23andMe test on campus or online and ship his/her saliva samples directly to 23andMe with a prepaid shipping label. The results of the 23andMe genotype test were delivered within 4–8 weeks (prior to the completion of the PCL course) through a free online 23andMe account to be accessed solely by the student for personal use. 23andMe provided participants with limited information regarding ancestry, carrier status, and genetic variability from the Illumina OmniExpress 23andMe v4 chip consisting of approximately 570,000 markers (San Diego, CA, USA). Students were instructed regarding use of the 23andMe website, including how to download raw data, but were also provided with demo profile information in case they opted out of the testing process. Because 23andMe presents results on a limited number of genes related to health, student pharmacists were referred to additional websites for detailed health information and provided with precautions and limitations of utilizing various online resources. The 23andMe test results were not directly linked to pharmacogenes; therefore, student pharmacists also had the option of extracting personal pharmacogenomic data from the 23andMe raw genotype file with a data-processing Excel spreadsheet developed by our lab using gene haplotype translation tables from PharmGKB for *CYP2C19*, *CYP2C9*, *CYP3A5*, *CYP2D6*, *DPYD*, *TPMT*, *G6PD*, *IFNL3*, *SLCO1B1*, and *VKORC1*. The data could be interpreted by using hyperlinked Clinical Pharmacogenetics Implementation Consortium (CPIC) guidelines within the spreadsheet. None of the individual pharmacogenomic results from the 23andMe tests were accessed, collected, or used in any of the teaching materials or for any research purpose.

Responses from the pre-intervention survey were collected for all student pharmacists to assess initial attitudes towards clinical pharmacogenomics. Data from students who completed both the pre- and post- intervention surveys was analyzed in a paired subset group and further delineated between those who underwent personal genotyping versus those who did not undergo personal genotyping. Students who responded to at least 70% of both the pre- and post-survey questions were included in the paired subset. McNemar’s test and the Wilcoxon signed rank test were used to analyze paired pre- and post-intervention survey responses respectively for binary comparisons and Likert items. Fisher’s exact test and the Mann Whitney-U test were used to analyze responses between genotyped and non-genotyped students respectively for binary comparisons and Likert items. Results were considered statistically significant if *p* < 0.05.

## 3. Results

### 3.1. Demographics

There were no statistically significant demographic differences between students who completed the pre-intervention survey and students in the paired subset group or between the genotyped and non-genotyped groups (Table 1). In the pre-intervention survey, the median age of pharmacy students was 24 years. The majority of students reported their ethnicity as white or Caucasian (68%) and having taken a previous genetics course (62%). Prior to the initiation of this study, only one student in the paired subset had pharmacogenomic testing performed in a medical setting. Thirty-one percent of students who completed the pre-intervention survey also completed the post-intervention survey, termed the paired subset. Fifty-five percent of students who completed both the pre-and post-educational interventions also obtained personal genotyping.

### 3.2. Educational Intervention

Table 2 and Table 3 summarize the differences between the paired subset overall, in addition to the differences between the genotyped and non-genotyped groups, after the educational intervention. In the post-educational intervention, 36% of students in the paired subset reported that they would recommend personal genotyping for a patient compared to just 19% before the pharmacogenomic intervention (*p* = 0.0032). Students were more confident (51% post-educational intervention versus 29% pre-educational intervention, *p* = 0.0045) in applying pharmacogenomic information to manage patients’ drug therapy. Overall after the educational intervention, more students (90% post-educational intervention versus 51% pre-educational intervention, *p* = 0.0072) believed that personal genomics will likely play an important role in their future career. There was a significant increase in the number of students who reported to be more familiar with pharmacogenomic resources for use in the clinical setting after the pharmacogenomic educational intervention regardless if they were genotyped (55% post-educational intervention versus 17% pre-educational intervention, *p* < 0.001) (Table 2). Students also became more confident in their ability to identify therapeutic areas in which pharmacogenomic testing is required (65% post-educational intervention versus 35% pre-educational intervention, *p* = 0.014) or recommended (75% post-educational intervention versus 45% pre-educational intervention, *p* = 0.027) (Table 3). Compared to the pre-intervention group, students became more confident post-educational intervention in their ability to interpret the results of pharmacogenomic testing from patients overall and within the genotyped groups (58% post-educational intervention versus 29% pre-educational intervention, *p* = 0.0029), but not within the non-genotyped groups (Table 2). More students reported that they could explain the rationale for pharmacogenomic testing in various therapeutic areas to patients (78% post-educational intervention versus 22% pre-educational intervention, *p* = 0.0074) (Table 3). Table A1 and Table A2 in the appendix lists additional findings between the paired subset and genotyped and non-genotyped groups after the educational intervention.

### 3.3. Personalized Genotyping

A greater percentage of individuals in the post-educational intervention genotyped group compared to the non-genotyped group agreed that information from a pharmacogenomic test may improve the way their medication treatment will be managed in the future (95% post-educational intervention genotyped versus 81% post-educational intervention non-genotyped, *p* = 0.0094) and would recommend the use of pharmacogenomic testing to manage patient therapy prospectively (82% post-educational intervention genotyped versus 52% post-educational intervention non-genotyped, *p* = 0.0259) (Table 2). Similarly, there was a significant difference in the belief that the pharmacy profession should be more active in educating patients and other healthcare providers about pharmacogenomics between the post-educational intervention genotyped and non-genotyped groups (82% post-educational intervention genotyped versus 65% post-educational intervention non-genotyped, *p* = 0.0392) (Table 3).

Table 4 and Table 5 contain results for students who elected to undergo personal genotyping. 76% reported that their learning experience was enhanced by doing so, and 71% claimed to have a better understanding of pharmacogenomics based on undergoing personal genotyping (Table 4). Additional post-intervention professional reflections and attitudes towards pharmacogenomics in the curriculum for all students who completed the post-intervention survey (regardless if they completed pre-intervention questionnaire and delineated by genotyped and non-genotyped groups) are reported in Table A3 in Appendix A.

## 4. Discussion

As pharmacy schools are the leaders and innovators that drive pharmacy practice forward, educational interventions should have a positive and beneficial impact to shape student thoughts and impressions of pharmacogenomics that will ultimately be carried with them into clinical practice. Because the implementation of pharmacogenomics is coming to fruition in the clinical setting [20,21,22,23,24,25,26,27,28], pharmacogenomics should be added to pharmacy school curricula. Education in pharmacogenomics helps pharmacy students understand the clinical utility and application of pharmacogenomics-guided therapeutic drug selection and adjustment. The educational intervention provided in this study was designed to make learning pharmacogenomics more engaging and experiential. Students demonstrated a significant increase in their confidence in applying clinical pharmacogenomic information and knowledge of clinical resources to manage patients’ drug therapy and were more likely to recommend personal genotyping for a patient.

This study was beneficial in demonstrating that pharmacogenomic educational interventions can make a difference to student attitudes in a way that could eventually lead to more acceptance of clinical pharmacogenomics in practice despite limitations. Limitations of this study include lack of a testing component to assess objective learning competencies. There was also a relatively low response rate due to optional survey participation, which may have introduced selection bias. This limitation may also be compounded by the evolution of the survey instrument. Originally adapted from Salari et al. [17,18], the survey instrument to assess attitudes and confidence had slight changes from year 1 to year 2 (e.g., additional items were added). These changes do provide us with additional insight into personalized genotyping, particularly as future studies will incorporate alternative assays focusing solely on pharmacogenes.

Additional schools of pharmacy are implementing personalized genotyping into the curriculum. Weitzel et al. introduced personalized genotyping into a pharmacogenomics elective [29]. Adams et al. described an initiative termed “Test2Learn” using 23andMe as their genotyping platform [30]. Most students felt pharmacogenotyping was an important part of the course and felt they had a better understanding of pharmacogenomics because of the genotyping activity. There are also limitations in personalized genotyping with 23andMe, as information on specific pharmacogenes is not readily available or interpretable. Future studies will examine the use of in-house next-generation sequencing assays that selectively examine pharmacogenes in the education of student pharmacists. Next-generation sequencing may provide some benefits compared to 23andMe in terms of potentially lower costs, greater flexibility, faster turnaround time to obtaining results, and more autonomy with an in-house assay compared to the use of a larger commercial company. With a next-generation sequencing assay, analysis of only pharmacogenes can occur, rather than examining genetic variants related to disease state and ancestry, in order to reduce ethical conundrums and enhance the relevance of the exercise to future pharmacists.

The study described here largely adhered to educating students on existing pharmacogenomics guidelines and applications. However, personalized medicine continues to transform practice. In the future, we may introduce additional facets of personalized medicine beyond pharmacokinetics, pharmacodynamics, and pharmacogenomics, including the effects of additional systems such as the microbiome and circadian rhythm [31,32,33]. As pharmacogenomic testing services become increasingly available to patients, either through healthcare providers or direct-to-consumer routes, there is more opportunity for pharmacists to provide pharmacogenomic counseling as an extension of medication-therapy-management (MTM) services. Pharmacists are ideally equipped to evaluate medication therapy challenges and implement solutions based on evidence-based precision medicine research. Effective pharmacogenomic educational interventions in PharmD curriculums can help pharmacy students better understand what a patient’s personal genotyping experience might be like and empower them to implement these valuable clinical services in their practice as future pharmacists.

## Figures and Tables

**Table 1:** Study population demographics and previous experience with clinical genetics. The number (and percentage) of students with each characteristic is reported. Note, sex was not collected in the pre-educational intervention for the first year.

Characteristics	Pre-Intervention (*n* = 222)	Paired Subset (*n* = 69)	23andMe Genotyped (*n* = 38)	Non-Genotyped (*n* = 31)
Median Age (Range)	24 (20–47)	24 (21–47)	24 (21–41)	24 (21–37)
Female		46 (67%)	26 (67%)	20 (65%)
Ethnicity				
Asian	39 (18%)	7 (10%)	4 (11%)	3 (7%)
Black or African American	14 (6%)	4(6%)	2 (8%)	2 (7%)
Hispanic or Latino	4 (2%)	3 (4%)	3 (8%)	0 (0%)
White or Caucasian (not Hispanic or Latino)	151 (68%)	53 (77%)	28 (74%)	25 (81%)
Level of Education				
Undergraduate coursework	49 (22%)	12 (17%)	7 (18%)	5 (16%)
Associate Degree	3 (1%)	1 (1%)	0 (0%)	1 (3%)
Bachelor Degree	155 (70%)	51 (74%)	28 (74%)	23 (74%)
Graduate Degree	12 (5%)	4 (6%)	3 (8%)	1 (3%)
Professional Degree	3 (1%)	1 (1%)	0 (0%)	1 (3%)
Past Genetics Course	137 (62%)	45 (66%)	22 (56%)	23 (64%)

**Table 2:** Personal and professional reflections and attitudes towards pharmacogenomics and personal genotyping. The number and percent of individuals who agree or strongly agree with the survey question are represented within the table below.

Survey Question	Pre-Intervention (*n* = 222)	Paired Subset (*n* = 69)			Genotyped (*n* = 38)			Non-Genotyped (*n* = 31)			Genotyped vs. Non-Genotyped Post
		Pre	Post	*p* Value	Pre	Post	*p* Value	Pre	Post	*p* Value	*p* Value
The information from a pharmacogenomic test may improve the way my medication treatment is currently managed.	187 (84%)	19 (28%)	52 (75%)	0.0473	34 (89%)	29 (76%)	0.0644	26 (84%)	23 (72%)	0.513	0.3541
The information from a pharmacogenomic test may improve the way my medication treatment will be managed in the future.	204 (92%)	66 (95%)	61 (88%)	0.1035	37 (97%)	36 (95%)	0.8167	29 (93%)	25 (81%)	0.109	0.0094
Pharmacogenomics is useful in managing drug therapy.	185 (83%)	59 (86%)	56 (81%)	0.4721	31 (82%)	32 (84%)	0.9528	28 (90%)	24 (77%)	0.292	0.5922
I am confident in my ability to understand the results of pharmacogenomic testing.	113 (51%)	28 (41%)	30 (43%)	0.4911	15 (39%)	20 (53%)	0.1102	13 (42%)	10 (32%)	0.478	0.1098
I am familiar with pharmacogenomic resources (e.g., guidelines) for use in the clinical setting.	55 (25%)	12 (17%)	38 (55%)	<0.0001	8 (21%)	22 (58%)	<0.0001	4 (13%)	16 (52%)	0.0002	0.7042
I would recommend the use of pharmacogenomic testing to manage therapy prospectively.	139 (63%)	44 (64%)	47 (68%)	0.2863	27 (71%)	31 (82%)	0.8724	17 (55%)	16 (52%)	0.21	0.0259
I am confident in applying pharmacogenomic information to manage patients’ drug therapy.	77 (35%)	20 (29%)	35 (51%)	0.0045	11 (29%)	22 (58%)	0.0029	9 (29%)	13 (42%)	0.305	0.3406
I know enough about genetics to understand personal genome test results.	108 (49%)	32 (46%)	41 (59%)	0.2151	18 (47%)	25 (66%)	0.1405	14 (45%)	16 (52%)	0.969	0.2631
Personal genomics will likely play an important role in my future career.	137 (62%)	35 (51%)	55 (90%)	0.0072	23 (61%)	32 (84%)	0.074	12 (39%)	23 (74%)	0.095	0.084
Most pharmacists have enough knowledge to help individuals interpret results of personal genome tests.	52 (23%)	13 (19%)	21 (30%)	0.9936	8 (21%)	11 (29%)	0.8978	5 (16%)	10 (32%)	0.912	0.731
Most people can accurately interpret their personal genome test results.	13 (6%)	4 (6%)	11 (16%)	0.7011	3 (17%)	5 (13%)	0.5786	1 (3%)	6 (19%)	0.274	0.1635
I would recommend a personal genotyping test for a patient at this time.	46 (21%)	13 (19%)	25 (36%)	0.0032	7 (18%)	14 (37%)	0.0923	6 (19%)	11 (35%)	0.017	0.3482

**Table 3:** Personal and professional reflections and attitudes towards pharmacogenomics and personal genotyping. The number and percent of individuals who agree or strongly agree with the survey question are represented within the table below. Only data from the second year is shown in the pre-educational intervention column; it was not collected in the pre-educational intervention for the first year.

Survey Question	Overall (Pre *n* = 29, Post *n* = 69)			Genotyped (Pre *n* = 16, Post *n* = 38)			Non-Genotyped (Pre *n* = 13, Post *n* = 31)			Genotyped vs. Non-Genotyped Post
	Pre	Post	*p* Value	Pre	Post	*p* Value	Pre	Post	*p* Value	*p* Value
I can explain the rationale for pharmacogenomic testing in various therapeutic areas to patients.	15 (52%)	54 (78%)	0.0074	11 (69%)	31 (82%)	0.1172	4 (31%)	23 (64%)	0.277	0.3293
I can identify therapeutic areas in which pharmacogenomic testing is required.	10 (35%)	45 (65%)	0.0138	7 (44%)	27 (71%)	0.1445	3 (23%)	18 (58%)	0.219	0.2786
I can identify therapeutic areas in which pharmacogenomic testing is recommended.	13 (45%)	52 (75%)	0.0268	10 (63%)	32 (84%)	0.1328	3 (23%)	20 (65%)	0.969	0.0263
I can interpret the results of pharmacogenomic testing from patients.	9 (31%)	33 (48%)	0.0305	6 (38%)	20 (53%)	0.0469	3 (23%)	13 (42%)	0.912	0.2651
The pharmacy profession should be more active in educating patients and other healthcare providers about pharmacogenomics.	22 (76%)	51 (74%)	0.8516	13 (82%)	31 (82%)	1	9 (69%)	20 (65%)	0.274	0.0392

**Table 4:** Reflections and attitudes towards personal genome testing for students who elected to undergo genotyping. The number and percent of individuals who agree or strongly agree with the survey question are represented within the table below.

Survey Question	Paired Subset Individuals Who Were Genotyped (*n* = 38)
My learning experience was enhanced by undergoing personal genotyping.	29 (76%)
I have a better understanding of pharmacogenomics on the basis of undergoing personal genotyping.	27 (71%)
Undergoing personal genotyping was an important part of my learning.	21 (55%)
This course helped me understand what a patient’s experience might be like if they chose to undergo personal genome testing.	32 (84%)
The cost for personal genome testing was reasonable.	33 (87%)
I would be willing to pay the full price (less than $100.00 plus shipping and handling) for personal genome testing.	6 (15%)
I was pleased with my decision regarding personal genome testing.	34 (89%)
I experienced anxiety when deciding whether to undergo personal genome testing.	6 (15%)
I experienced anxiety when awaiting my personal genome testing results.	5 (13%)
I experienced anxiety after receiving my personal genome testing results.	1 (2%)
The opportunity to ask healthcare professional for help in interpreting the results is an important component to a personal genome testing offer.	31 (81%)

**Table 5:** Reflections and attitudes towards personal genome testing for students who elected to undergo genotyping. The number and percent of individuals who agree or strongly agree with the survey question are represented within the table below. Only data from the second year is shown; it was not collected in the post-educational intervention for the first year.

Survey Question	Paired Subset Individuals Who Were Genotyped (*n* = 16)
The personal genome testing experience was favorable.	15 (94%)
The information received from personal genome testing was easy to understand.	10 (63%)
The information received from personal genome testing was misused, mishandled, or misinterpreted.	1 (6%)
The information received from personal genome testing will be helpful when making clinical decisions in the future.	9 (56%)

## Appendix A

### Educational Intervention Details

During week 8 of the 1-h large student group session of PCL, student pharmacists completed a pre-intervention survey before observing an introductory lecture on pharmacogenomics. In this same lecture, students received information regarding voluntary, anonymous personal genomic testing through 23andMe (Mountain View, CA, USA). The large group lecture included the following student learning objectives: define pharmacogenomics, discuss the importance of pharmacogenomics in drug therapy, examine how pharmacogenomics is used to manage drug therapy, and provide examples of pharmacogenomic-guided algorithms.

During week 11 in the small group sessions of PCL, pharmacogenomic patient cases using demo 23andMe data were discussed and relevant pharmacogenomic clinical resources were reviewed. These clinical resources were evidence-based and included CPIC guidelines and the PharmGKB website. A final lecture was given in week 15 of PCL to the large, complete student group with the following student learning objectives: discuss 23andMe results, demonstrate how to obtain pertinent pharmacogenomic information and utilize online resources, and review a clinical case focusing on the use of pharmacogenomics to manage drug therapy. Throughout the educational intervention, eight different drug-gene pairs were described using clinical cases. After the wrap-up lecture in week 15, students were asked to complete the post-intervention survey.

**Table A1:** Additional personal and professional reflections and attitudes towards pharmacogenomics and personal genotyping. Items assessed on a five-point Likert scale are presented as the number and percentage of student pharmacists agreeing or strongly agreeing with the corresponding statement.

Survey Question	Pre-Intervention (*n* = 222)	Paired Subset (*n* = 69)			Genotyped (*n* = 38)			Non-Genotyped (*n* = 31)			Paired Subset Genotyped vs. Non-Genotyped
		Pre	Post	*p* Value	Pre	Post	*p* Value	Pre	Post	*p* Value	*p* Value
I am comfortable with the use of my pharmacogenomic information to guide clinicians in selecting the appropriate medication for me.	161 (73%)	50 (72%)	51 (74%)	0.8625	28 (74%)	30 (79%)	0.8439	22 (71%)	21 (68%)	1	0.4504
I am comfortable with the use of my pharmacogenomic information to guide clinicians in selecting the appropriate dose of my medication.	159 (72%)	50 (72%)	48 (70%)	0.3781	29 (76%)	28 (74%)	0.3533	21 (68%)	20 (65%)	0.79	0.5757
Pharmacogenomic information should be stored in the patient’s medical record.	169 (76%)	56 (86%)	56 (81%)	0.4672	30 (79%)	31 (82%)	0.9542	26 (84%)	25 (81%)	0.285	0.3507
Pharmacogenomics will likely play an important role in my future career.	165 (74%)	53 (77%)	57 (83%)	0.657	31 (82%)	34 (89%)	0.9896	22 (71%)	23(74%)	0.689	0.438
Most physicians have enough knowledge to help individuals interpret results of personal genome tests.	48 (22%)	10 (14%)	19 (28%)	0.7498	5 (13%)	10 (26%)	0.8294	5 (16%)	9 (29%)	0.877	0.5478
Personal genome testing companies provide an accurate analysis and interpretation of genotype data.	46 (21%)	14 (20%)	22 (32%)	0.7314	9 (25%)	12 (32%)	0.958	5 (16%)	10 (32%)	0.804	0.6758
Personal genome testing companies should be regulated by the federal government (i.e., the Food and Drug Administration).	129 (58%)	39 (57%)	41 (59%)	0.9315	22 (58%)	23 (61%)	0.6575	17 (55%)	18 (58%)	0.705	0.7559

**Table A2:** Additional personal and professional reflections and attitudes towards pharmacogenomics and personal genotyping. Items assessed on a five-point Likert scale are presented as the number and percentage of student pharmacists agreeing or strongly agreeing with the corresponding statement. Only data from the second year is shown; it was not collected in the pre- or post-educational intervention for the first year.

Survey Question	Paired Subset (*n* = 29)			Genotyped (*n* = 16)			Non-Genotyped (*n* = 13)			Paired Subset Genotyped vs. Non-Genotyped
	Pre	Post	*p* Value	Pre	Post	*p* Value	Pre	Post	*p* Value	*p* Value
I understand the risks of using personal genome testing services.	8 (28%)	13 (45%)	0.4034	6 (38%)	8 (50%)	1	2 (15%)	5 (38%)	0.277	0.9634
I understand the benefits of using personal genome testing services.	15 (52%)	23 (79%)	0.2056	10 (63%)	13 (82%)	0.6172	5 (33%)	10 (77%)	0.219	0.5685
I can discuss the risks of pharmacogenomic testing with patients.	6 (21%)	11 (38%)	0.328	4 (25%)	9 (56%)	0.1582	2 (15%)	2 (15%)	0.095	0.0584
I can discuss the benefits of pharmacogenomic testing with patients.	17 (57%)	25 (86%)	0.0783	11 (69%)	14 (88%)	0.1875	6 (46%)	11 (85%)	0.877	0.5711

**Table A3:** Post-intervention professional reflections and attitudes towards pharmacogenomics in the curriculum in genotyped versus non-genotyped groups.

Survey Question	Genotyped (*n* = 53)	Non-Genotyped (*n* = 55)	*p*-Value
The Pre-Pharmaceutical Care Lab lecture enhanced my learning of pharmacogenomics.	39 (74%)	36 (65%)	0.5035
The cases in Pharmaceutical Care Lab enhanced my learning of pharmacogenomics.	35 (66%)	34 (62%)	0.6653
The supplementary class materials for interpreting personal pharmacogenomic results are useful.	34 (64%)	27 (49%)	0.472
The supplementary class materials for additional personal genome testing results are useful.	35 (66%)	29 (55%)	0.3158
More time should be spent on pharmacogenomics material in Pharmaceutical Care Lab.	17 (32%)	8 (15%)	0.9278
More time should be spent on pharmacogenomics material in the curriculum.	20 (38%)	18 (33%)	0.4547
A separate pharmacogenomics course should be required in the curriculum.	12 (23%)	9 (16%)	0.8097
An elective pharmacogenomics course should be available in the curriculum.	48 (91%)	39 (71%)	0.1583
Pharmacogenomics should be covered early in the curriculum prior to therapeutic coursework.	24 (45%)	11 (20%)	0.4202
Pharmacogenomics should be covered as needed in therapeutic coursework.	48 (91%)	42 (76%)	0.0135
Pharmacogenomics should be covered in practical clinical coursework.	41 (77%)	31 (56%)	0.6082
Pharmacogenomics cases should be incorporated into coursework.	37 (70%)	26 (47%)	0.0384
